# Reduced period from follicular wave emergence to luteolysis generated greater steroidogenic follicles and estrus intensity in dairy cows

**DOI:** 10.1038/s41598-023-50001-x

**Published:** 2023-12-20

**Authors:** T. Minela, P. Gibb, S. McBeth, A. Santos, J. R. Pursley

**Affiliations:** Department of Animal Science, Michigan State University, East Lansing, MI USA

**Keywords:** Endocrine reproductive disorders, Infertility

## Abstract

The onset of productive life in dairy cattle, concomitant to parturition, is accompanied by a substantial decrease in fertility in comparison with non-lactating, nulliparous heifers. Follicular growth patterns differ between parous and nulliparous dairy cattle. Nulliparous heifers ovulate follicles with reduced antral age (RAA). This study aimed to exogenously reduce ovulatory follicle age in lactating dairy cows from 7 to 5 days old. Cows (n = 80) had their estrous cycles synchronized with the Double-Ovsynch program. At the final portion of this program, luteolysis was induced at either 5 (RAA) or 7 (Control) days following follicular wave emergence. RAA outcomes were estimated in comparison with Controls. RAA resulted in smaller follicles 2 days post-treatment. Despite lower serum concentrations of 17β-estradiol before treatment compared with Controls, the rate of increase in this hormone was greater for the RAA treatment. There was no difference in luteolysis rates between treatments. Proestrus (luteolysis onset to estrus onset) was prolonged in RAA cows. Cows with RAA had more intense estruses. Collectively, these results indicate that decreasing the age of the ovulatory follicle may improve the steroidogenic capacity of the dominant follicle and estrus expression intensity in lactating dairy cows.

## Introduction

The transition from nulliparous heifer to lactating cow comes with high costs to fertility^1^. The chance of pregnancy decreases 50% in addition to significant reductions in the behavioral ability of cows to exhibit estrus. Significant changes in ovarian function occur during this transition. Nulliparous heifers have predominantly three follicular waves of follicle development during an estrous cycle^2^. After their first calving, these newly lactating cows have primarily two waves of follicular development culminating with an ovulatory follicle that has undergone several more days of development compared with heifers with three follicular waves. The time from emergence of the second follicular wave in cows can be variable between 9 and 14 days of the cycle^3^. The third follicular wave of development in heifers begins around day 16 of the cycle^4^. Considering heifers have a shorter estrous cycle compared with lactating cows^2^, the differences in antral age of the ovulatory follicle may be a reason for the differences in fertility between these two groups.

Follicular steroidogenic potential may be altered with longer or shorter periods of antral follicle development. Intrafollicular fluid concentrations of estradiol increased exponentially with follicle diameter in both cows^5^ and heifers^6^. Nonetheless, in at least two studies^2,7^ heifers had greater concentrations of 17β-estradiol (E_2_) prior to ovulation compared with cows, despite having ovulatory follicles of smaller diameter^7^. The period between follicular emergence and estrus was also shorter in heifers compared with cows^2^. This could suggest that shortening duration of follicular development in lactating cows to replicate what naturally occurs in heifers may impact endocrine function of the pre-ovulatory follicle and estrus expression.

In an unpublished study from our laboratory, different lengths of time from follicular wave emergence to luteolysis were studied to determine differences in follicular dynamics. These data indicated that follicles with a dominance period of 5 days grew at faster rate from follicular wave emergence to the luteinizing hormone (LH) surge (1.9 ± 0.04 mm/day) and had a greater peak of estradiol following induced luteolysis (2.6 ± 0.3 pg/mL) compared with periods of 6 (1.7 ± 0.05 mm/day, and 2.0 pg/mL) or 7 days (1.6 ± 0.05 mm/day, and 2.1 pg/mL) from wave emergence.

Fertility programs were developed to increase pregnancies per artificial insemination (AI) in lactating dairy cows utilizing timed-AI. These programs limit the antral age of the ovulatory follicle compared with natural estrus via manipulation of ovarian function to be more like a heifer with three waves of follicular growth. Gonadotropin releasing hormone (GnRH) and prostaglandin F_2α_ (PGF_2α_) pharmaceutical analogues are commonly utilized for synchronization of ovulation in lactating dairy cows. Combining the precise timing of GnRH and PGF_2α_ to induce follicular wave emergence, growth of a new dominant follicle, luteolysis, and ovulation within an 8-h period allowed for AI at the most ideal stage of lactation. This method was first described in 1995, by Pursley and collaborators^8^, and named Ovsynch. Ovsynch, and its variations^9–11^, also improve fertility via manipulation of corpora lutea function and antral age of the ovulatory follicle. Double-Ovsynch^9^ is widely utilized for first post-partum AI to improve fertility of dairy cows compared with AI following a detected estrus and is the gold standard for control of follicle and corpus luteum function in dairy cows^12^. Minela et al. ^13^ reported that 92% of lactating dairy cows had ovulation and induction of a new follicular wave following the first GnRH of the second Ovsynch of Double-Ovsynch. This allowed 7 days of development prior to the PGF_2α_ induced luteolysis. Luteolysis can be induced on either day 5 or 7 of follicular development in both beef^14^ and dairy^15^ cattle, hence shortening or prolonging the antral age of the pre-ovulatory follicle prior to AI. Pregnancies per AI^16^ and embryo quality^17^ were both improved in lactating dairy cows when follicular dominance was shortened. Final follicular and oocyte maturation events take place following luteolysis (proestrus phase of the estrous cycle)^18,19^. Longer proestrus length was associated with greater estrus expression prior to timed-AI and greater pregnancies per AI in beef heifers^20^.

Estradiol secreted by the preovulatory follicle triggers external expression of sexual receptivity^21,22^. The primary sign of estrus in cattle is the act of standing to be mounted by another cow. This behavior can be observed in only ~ 50% of dairy cows, following twice a day visual observation^1^. Electronic estrus detection systems are utilized in beef and dairy operations to improve estrus detection rates (~ 20% increase compared with visual detection)^23^. These systems utilize secondary estrus characteristics, such as increase in activity (deviation from a 7-day baseline) and the level/intensity of estrus. The duration of follicular development, follicular characteristics, and endocrine output may impact these characteristics as detected by automated activity monitors.

Estrus expression around timed-AI was associated with increased probability of pregnancy^24^. Supplementation of E_2_ analogues at the final GnRH of Ovsynch increased the estrus expression^25^. Madureira et al. demonstrated that not just estrus expression around timed-AI, but level of estrus intensity, increased the chances of pregnancy of lactating dairy cows. Occurrence of estrus around AI modified gene expression in both the endometrium and conceptus 19 days post-AI compared with cows with no estrus but with ovulation and normal luteal development^26^. These changes were associated with genes involved in the pre-attachment phase of conceptus development^27^. Moreover, antral follicular age was associated with pregnancy status following AI to a natural estrus. Cows with established pregnancy 35 days after AI had almost one less day of follicular development (emergence to estrus; 7.8 ± 0.2 days) compared with non-pregnant cows (8.6 ± 0.2 days), regardless of number of follicular waves^28^. Thus, antral age of the pre-ovulatory follicle at time of the luteinizing hormone (LH) surge may play a key role in the fertility potential of the oocyte within that follicle^17^.

**Figure 1:**
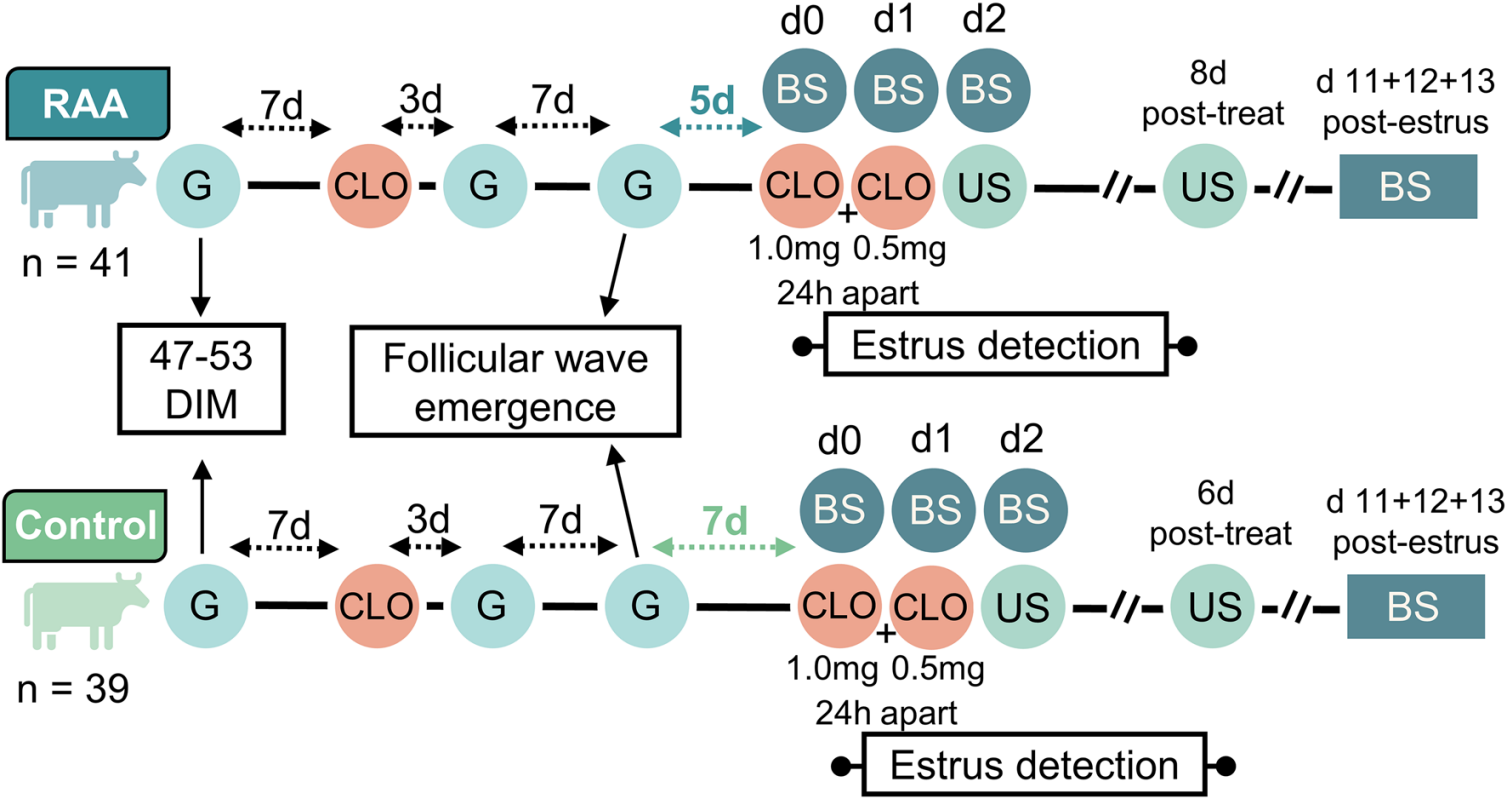
Experimental design to determine the effects of shorter (RAA) vs longer (Controls) development periods of ovulatory follicles utilizing the fertility program Double-Ovsynch in lactating Holstein cows that were detected, or not, in estrus. Double-Ovsynch was initiated between 47–53 days in milk (DIM) for both treatments (treat). Gonadotropin-releasing hormone (G) was utilized to induce follicular wave emergence in both treatments. Estrus detection was performed with automated activity monitors following cloprostenol sodium (CLO) first administered on day 0. Blood samples (BS) were collected on day (d) 0, 1, 2, from CLO, and at day 11, 12 and 13 after a detected estrus. Ultrasound (US) was utilized to measure follicular diameter at day 2 post-induction of luteolysis and to confirm ovulation on days 6 or 8 post-induction of luteolysis.

The objective of this study was to reduce the period from follicular wave emergence to induced luteolysis (i.e., reduce the ovulatory follicle antral age) to test the effects on follicular, endocrine and estrus characteristics in lactating dairy cows. Our novel design allowed for lactating dairy cows to have unadulterated maturation of the ovulatory follicle to gain a greater understanding of the interaction of antral age of the ovulatory follicle at luteolysis and steroidogenic capacity. We hypothesized that reducing time from follicular wave emergence to the induction of luteolysis would result in (1) smaller pre-ovulatory follicles, (2) greater E_2_ concentrations following induced luteolysis, and (3) greater intensity and longer periods of estrus. If true, the outcomes could lead to alterations in synchronization programs that may improve fertility of lactating dairy cows. To test this hypothesis, cows were randomly assigned to treatments described in Fig. 1.

## Results

### Shortening the duration of follicular development resulted in smaller more steroidogenic follicles and equally steroidogenic corpora lutea post-ovulation

**Figure 2:**
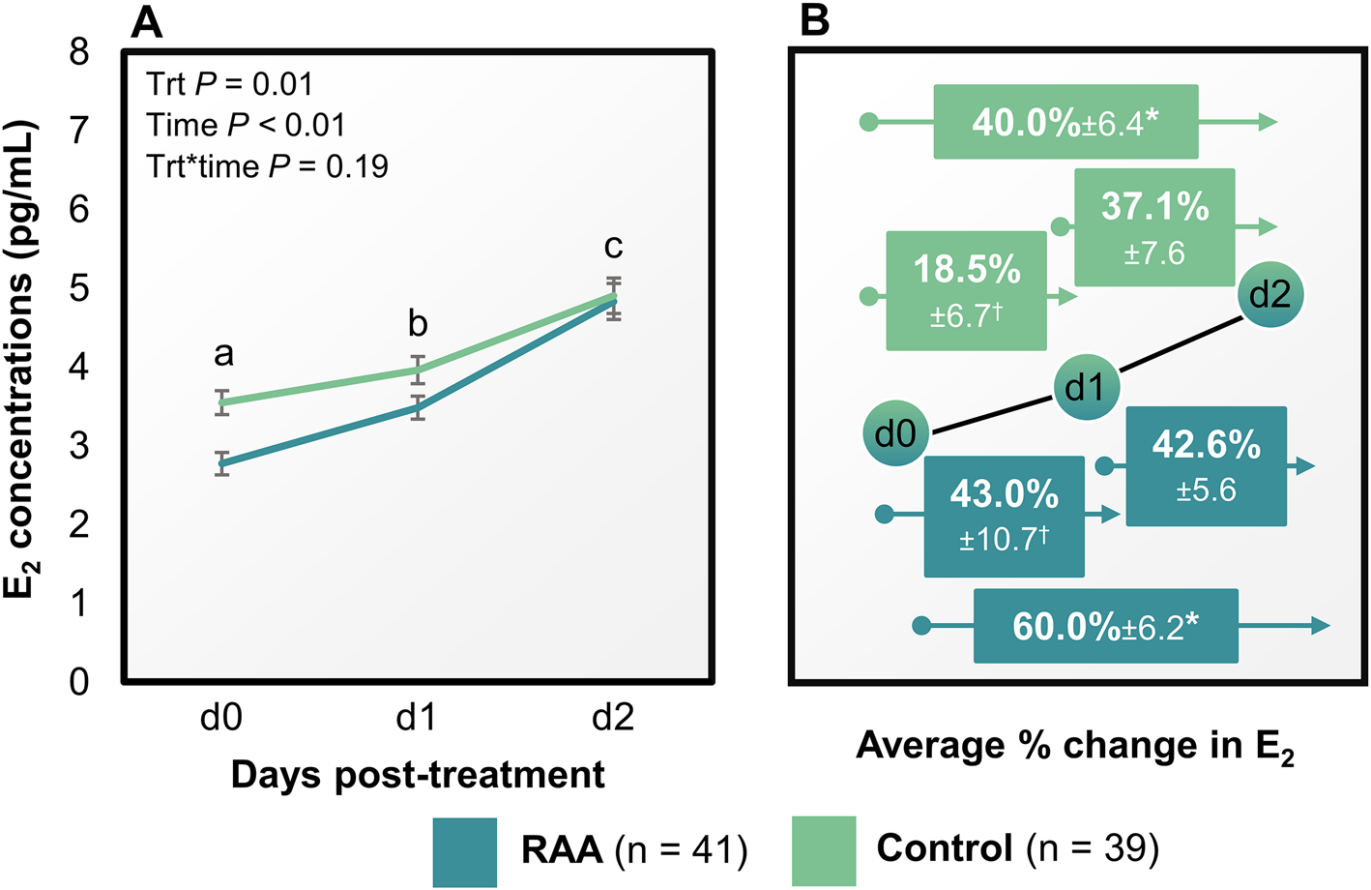
Effect of treatment on 17β-estradiol (E_2_) concentrations (pg/mL) in lactating Holstein cows following cloprostenol sodium at day (d) 5 (reduced antral age; RAA) or 7 (Control) of follicular development (**A**). Illustration of the percentage change in E_2_ concentrations between days 0 and 1, 1 and 2, and days 0 and 2 in lactating Holstein cows following cloprostenol sodium at day 5 or 7 of follicular development (**B**). The letter superscript describes the comparison between treatments within day post-induction of luteolysis. The letters “a” and “b” denote a *P* ≤ 0.01, and “c” denotes a *P* = 0.15. *Indicates a greater % increase in E_2_ concentrations between day 0 and 2 in RAA compared with Controls (*P* = 0.03). ^†^Indicates a tendency for greater % increase in E_2_ concentrations between day 0 and 1 in RAA in comparison with Controls (*P* = 0.08). The % increase in E_2_ concentrations between day 1 and 2 was not different between treatments (*P* = 0.53). Data are shown as means ± SEM.

Reducing the days between follicular emergence to onset of luteolysis (reduced antral age; RAA) effectively resulted in smaller single dominant follicles measured 2 days post-luteolysis in comparison with Controls (13.5 ± 0.4 vs 15.1 ± 0.4 mm diameter; *P* = 0.01). There was an effect of treatment on concentrations of E_2_ between day 0 and 2 post-induction of luteolysis (*P* < 0.01; Fig. 2A). Controls had greater E_2_ concentrations at days 0 and 1 in comparison with RAA (*P* ≤ 0.01). However, there were no detectable differences in E_2_ concentrations at day 2 post-induction of luteolysis (*P* = 0.15). The rate of increase of E_2_ concentrations, as a marker of the dominant follicle secretory potential, was greater for RAA compared with Controls between days 0 and 2 post-induction of luteolysis, despite smaller follicular diameter (*P* = 0.01; Fig. 2B). Previously unpublished data from our laboratory indicated a greater rate of increase in E_2_ concentrations (measured every 12 h) in day 5 follicles vs day 7.

**Figure 3:**
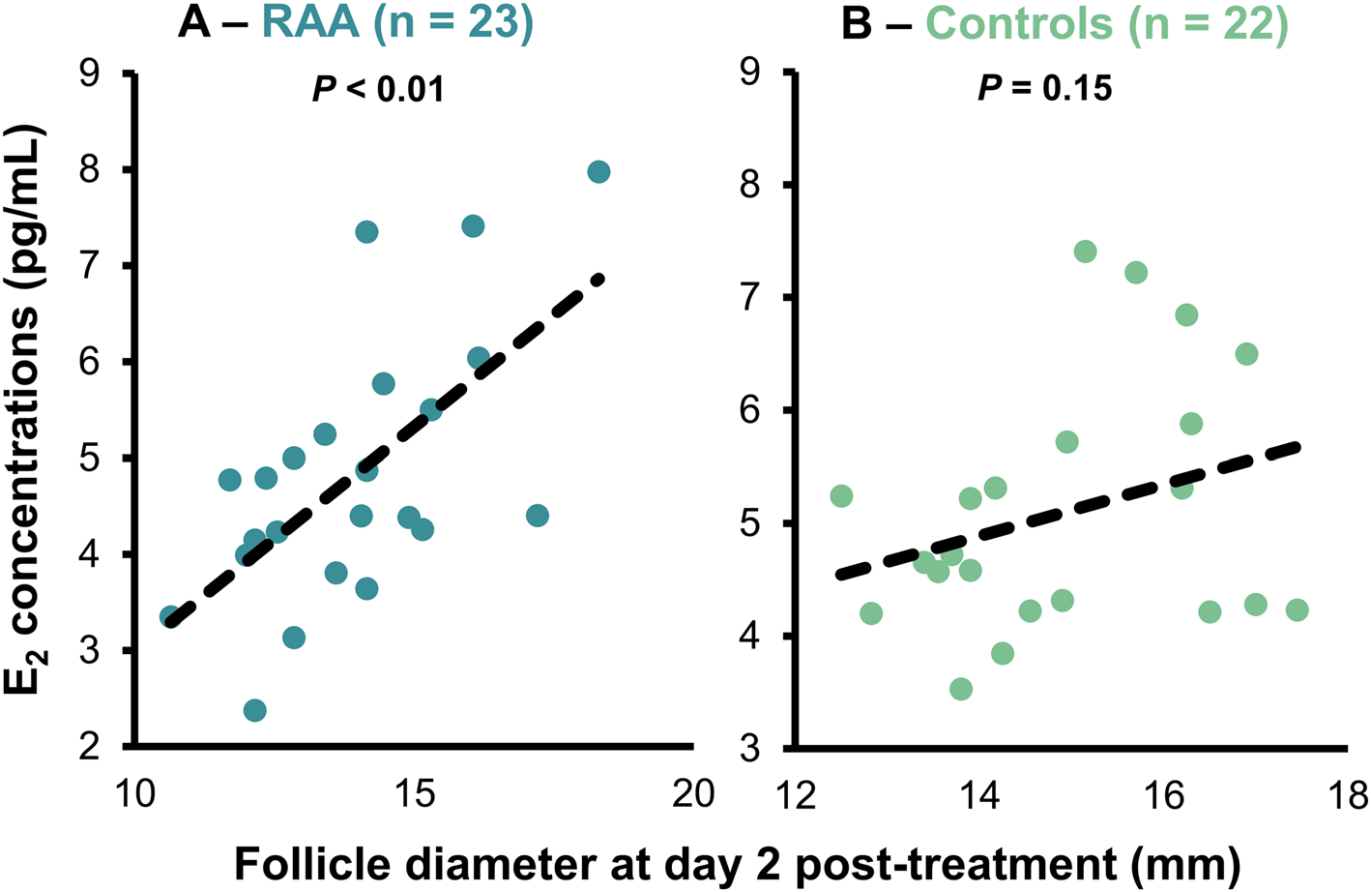
Linear relationship within treatment between 17β-estradiol (E_2_) concentrations (pg/mL) and follicle diameter (mm) in lactating Holstein cows 2 days post-induction of luteolysis with cloprostenol sodium at (**A**) day 5 (reduced antral age; RAA) or (**B**) day 7 (Control) of follicular development. Linear regression was estimated only in cows with single ovulations to isolate the relationship between the dominant follicle diameter and its E_2_ output.

Follicle diameter in cows with a single ovulation was linearly associated with E_2_ concentrations in RAA but not Controls, 2 days post-induction of luteolysis (Fig. 3A,B, respectively). Correlation coefficients were also estimated between E_2_ concentrations (days 0, 1, and 2) and follicle diameter 2 days after induction of luteolysis. In this case, there was a tendency for a positive correlation between E_2_ concentrations at day 0 and follicle diameter (r = 0.35; *P* = 0.06) in the RAA treatment. There was a moderately positive correlation between follicle diameter and E_2_ concentrations at day 1 (r = 0.76; *P* < 0.01) and 2 post-induction of luteolysis (r = 0.65; *P* < 0.01) in the RAA treatment. There was a negative correlation between follicle diameter and E_2_ concentrations at day 0 (r = − 0.43; *P* = 0.01), in Control cows. On days 1 and 2, there were no significant correlations between E_2_ concentrations and follicle diameter in Control cows (r = 0.27 and r = 0.26, respectively; *P* ≥ 0.13,).

Mid-cycle P_4_ concentrations (average concentrations on days 11, 12 and 13 post-estrus) did not differ between treatments in cows detected in estrus with confirmed ovulations (RAA 5.7 ± 0.6 vs Controls 5.3 ± 0.6 ng/mL; *P* = 0.68). Cows with double ovulation tended to have greater mid-cycle P_4_ concentrations in comparison with cows with single ovulations (6.7 ± 1.1 vs 5.0 ± 0.4 ng/mL, respectively; *P* = 0.08), but no interaction with treatment was detected (*P* = 0.24). Overall, follicular diameter at day 2 post-induction of luteolysis was not correlated with early mid-cycle P_4_ concentrations in cows with single ovulations (r = − 0.03, *P* = 0.83).

### The experimental design allowed for complete luteolysis in both treatments

**Figure 4:**
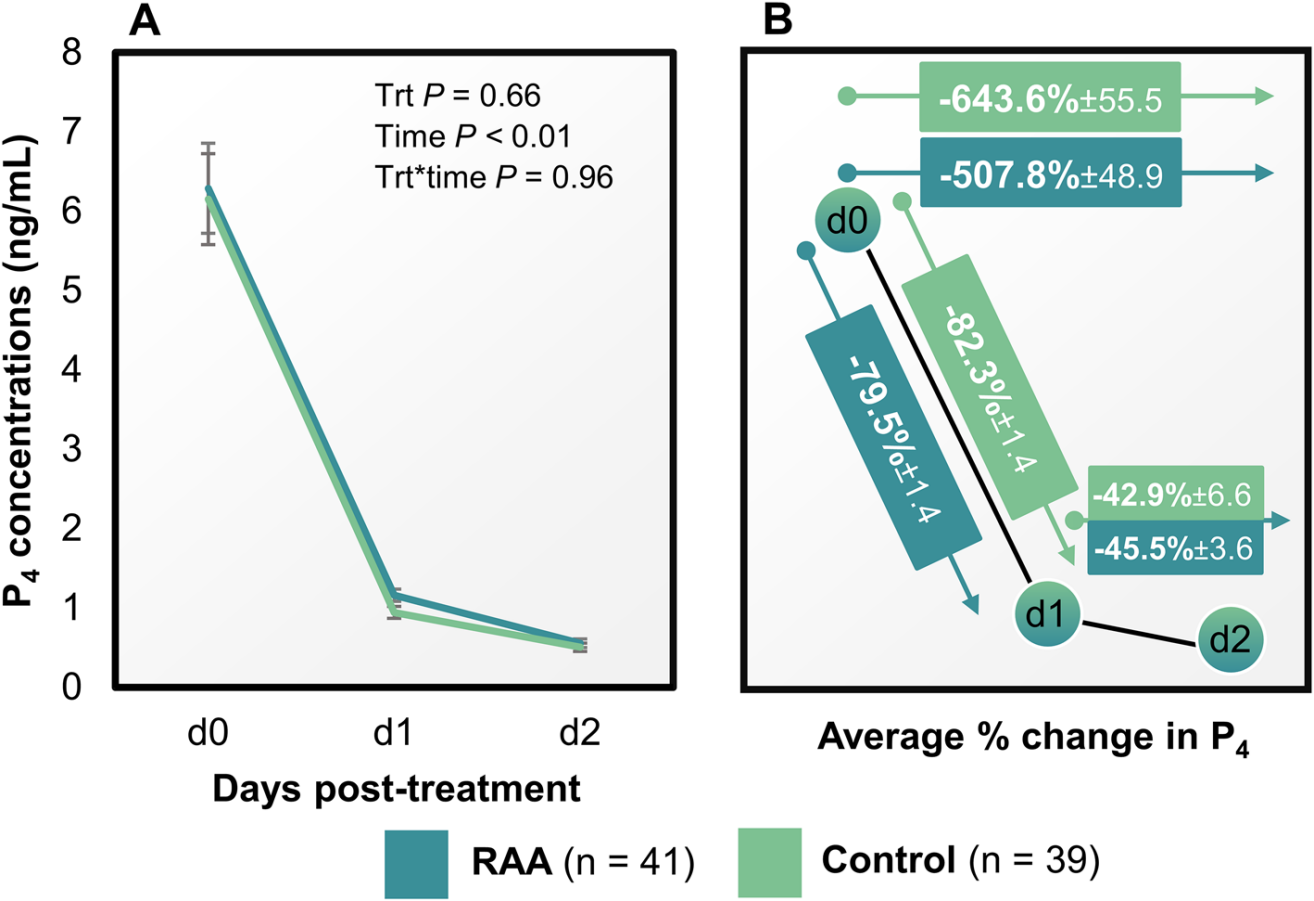
Effect of reducing the time from follicular wave onset to cloprostenol sodium (CLO) in lactating Holstein cows on progesterone (P_4_) concentrations (ng/mL) 0, 1 and 2 days (d) following induction of luteolysis with CLO (**A**). Illustration of the effect of treatment on percentage change in P_4_ concentrations between days 0 and 1, 1 and 2, and days 0 and 2 in lactating Holstein cows following CLO at day 5 (reduced antral age; RAA) or 7 (Control) of follicular development (**B**). No differences were observed in the % decrease in P_4_ concentrations between day 0 and 1, day 1 and 2, and day 0 and 2 (*P* ≥ 0.25) between treatments. Data are shown as means ± SEM.

Initial induction of luteolysis with 1 mg cloprostenol sodium at time of induction of luteolysis in addition to 0.5 mg cloprostenol sodium one day later induced complete luteal regression in 100% of the cows in both treatment groups. There was no effect of shortening the period from follicular emergence to onset of luteolysis on P_4_ concentrations at any time (*P* = 0.68; Fig. 4A). Rate of decrease in P_4_ between days 0 and 1, 1 and 2, and days 0 and 2 were not different amongst treatments (Fig. 4B; *P* ≥ 0.25).

### Concentrations of E_2_ were differentially impacted by treatment and double ovulations

Double ovulation occurred in 11/41 and 7/39 cows in RAA and Controls, respectively (*P* = 0.40). The effects of single or double ovulations within treatments on E_2_ concentrations and on the rate of increase in E_2_ are described in Table 1. Concentrations of E_2_ were greatest in Controls with double ovulations at days 0 and 1 (*P* ≥ 0.01), but no differences were detected at day 2 post-induction of luteolysis (*P* ≥ 0.35). The rate of increase in E_2_ between days 0 and 2 tended to be lower in Controls in comparison with RAA, in cows with double ovulations (*P* = 0.097).

**Table 1:** The effect of number of ovulations (ov.) within reduced antral age (RAA) and Control treatments on serum concentrations of 17β-estradiol (E_2_; pg/mL) and on the % change in E_2_ concentrations. Analyses were performed in cows with confirmed single or double ovulations within day, or periods post-induction of luteolysis. Multiple comparisons were only performed in the presence of significant two-way interactions. Letter superscript describes the comparison within day post-induction of luteolysis and between treatment and number of ovulation. Different letter superscripts denote a *P* ≤ 0.04. *Denotes a tendency of *P* = 0.06 for the comparison of RAA single ovulation vs Control single ovulation on day 0 post-induction of luteolysis. ^†^Denotes a tendency of *P* = 0.097 for the comparison of RAA double ovulation vs Control double ovulation on the period between day 0 to 2 post-induction of luteolysis. Data are shown as means ± SEM.

	RAA single ov. n = 23	RAA double ov. n = 11	Control single ov. n = 22	Control double ov. n = 7	Two-way interaction *P*-value^1^
E_2_ concentrations (pg/mL)					
Day 0	2.8 ± 0.2^a^*	2.6 ± 0.2^a^	3.3 ± 0.1^a^*	4.5 ± 0.5^b^	0.01
Day 1	3.5 ± 0.2^a^	3.7 ± 0.2^a^	3.8 ± 0.2^a^	5.0 ± 0.4^b^	0.047
Day 2	4.8 ± 0.3	5.1 ± 0.5	5.1 ± 0.2	5.9 ± 0.8	NS
% Change in E_2_					
Day 0 to 1	42.5 ± 15.7	49.3 ± 13.0	18.8 ± 9.4	19.9 ± 17.4	NS
Day 1 to 2	42.3 ± 8.3	39.4 ± 8.8	45.9 ± 12.4	17.7 ± 8.3	NS
Day 0 to 2	57.0 ± 8.1^a^	67.2 ± 10.9^a†^	50.7 ± 7.9^a^	23.6 ± 15.3^a†^	0.09

^1^Two-way interaction between number of ovulation and treatment.

*NS* non-significant and denotes a *P* ≥ 0.14.

### Treatments and parity impacted the proportion of cows with behavioral estrus

Overall, 73% (58/80) of cows were detected in estrus during the 8-day period after induction of luteolysis. There was a tendency for a greater percentage of cows in estrus with RAA compared with Controls (80.5 vs 64.1%, *P* = 0.09). Second parity cows had reduced chances of estrus behavior (55.6%) compared with first (80%; *P* = 0.04) and third-plus parity cows (81.2%; *P* = 0.05).

### Manipulating the period of follicular development influenced key estrus characteristics

Table 2 summarizes the main effects of estrus characteristics assessed with automated activity monitors. Cows in RAA had a reduced period between follicular wave emergence and onset of estrus compared with Controls. Additionally, RAA extended the proestrus period compared with Controls. Length of time in estrus, and time to peak activity did not differ between treatments (*P* ≥ 0.57). However, estrus intensity, measured as activity peak, was greater for RAA compared with Controls (*P* = 0.02). There was no significant correlation between E_2_ concentrations at day 0 and estrus intensity (r = 0.25; *P* = 0.17) in RAA cows. Concentrations of E_2_ at day 1 (r = 0.34; *P* = 0.05) and 2 (r = 0.32; *P* = 0.07) post-induction of luteolysis tended to be correlated with estrus intensity in RAA. There was no correlation between estrus intensity and E_2_ concentrations in Control cows (r = − 0.02, r = 0.08, and r = − 0.03, for days 0, 1, and 2, respectively; *P* ≥ 0.71).

**Table 2:** Effect of different intervals to induced luteolysis on estrus characteristics of lactating Holstein cows detected in estrus with automated activity monitors. Reduced antral age (RAA) treatment consisted of a 5-day period between follicular wave emergence and induced luteolysis with cloprostenol sodium (CLO). Controls had a 7-day period between follicular wave emergence and induced luteolysis with CLO. Onset of estrus was defined as the time of increased activity (≥ 35% change) in comparison with a cow 7-day mean activity. All cows that exhibited estrus were included in the analyses, regardless of ovulation status. Data are shown as means ± SEM.

	RAA (n = 33)	Control (n = 25)	*P*-value
Period from GnRH to estrus (days)^1^	8.5 ± 0.1	10.2 ± 0.1	< 0.01
Proestrus length (hours)^2^	89.4 ± 2.8	81.2 ± 3.5	0.01
Estrus length (hours)^3^	14.7 ± 0.6	14.0 ± 1.0	NS
Time to reach peak activity (hours)^4^	5.5 ± 0.6	5.4 ± 0.8	NS
Estrus intensity^5^	94.6 ± 1.2	86.8 ± 3.1	0.03

*NS* non-significant and denotes a *P* ≥ 0.37.

^1^Period between GnRH induction of a new follicular wave and estrus onset.

^2^Period between induction of luteolysis (first treatment with CLO) and estrus onset.

^3^Period between estrus onset to re-establishment of normal activity.

^4^Period between estrus onset and the highest/peak value of activity change observed during the estrus event.

^5^Highest/peak value of percentage activity change observed during the estrus event.

### Double ovulations had an impact on estrus characteristics

The effects of double ovulation and the interaction between treatment and double ovulation are described in Table 3. Cows in the RAA treatment had no differences in time to exhibit estrus from the day of follicular wave induction, regardless of double or single ovulations (*P* = 0.35). However, Control cows with double ovulation had shortened periods to estrus onset in relation to induction of follicular wave in comparison with Control cows that had a single ovulation (*P* < 0.01). Cows with double ovulation had diminished proestrus length in comparison with cows with a single ovulation (*P* < 0.01), but no interaction with treatment (*P* = 0.17). Estrus length, time to reach peak activity, and estrus intensity were no different between cows with double in comparison with single ovulations (*P* ≥ 0.11), with no treatment interaction (*P* ≥ 0.20).

**Table 3:** Effect of double ovulation (ov.) and the interactions between treatment and double ovulation on estrus characteristics of lactating Holstein cows detected in estrus with automated activity monitors. Reduced antral age (RAA) treatment consisted of a 5-day period between follicular wave emergence and induced luteolysis with cloprostenol sodium (CLO). Controls had a 7-day period between follicular wave emergence and induced luteolysis with CLO. Onset of estrus was defined as the time of increased activity (≥ 35% change) in comparison with a cow 7-day mean activity. Multiple comparisons were only performed in the presence of significant two-way interactions. Different letter superscripts denote a *P* ≤ 0.01. Only cows that exhibited estrus with confirmed single or double ovulations were included in the analyses. Data are shown as means ± SEM.

	Double ov. n = 16	Single ov. n = 40	RAA single ov. n = 21	RAA double ov. n = 10	Control single ov. n = 19	Control double ov. n = 6
Period from GnRH to estrus (days)^1^	8.7 ± 0.2	9.5 ± 0.2	8.7 ± 0.1^ac^	8.2 ± 0.1^a^	10.5 ± 0.1^b^	9.3 ± 0.2^c^
*P*-values	< 0.01		Two-way interaction: 0.04			
Proestrus length (hours)^2^	70.8 ± 3.7	91.1 ± 2.3	92.9 ± 3.3	77.5 ± 4.0	87.7 ± 3.2	60.7 ± 4.5
*P*-values	< 0.01		Two-way interaction: 0.17			
Estrus length (hours)^3^	13.5 ± 0.8	14.8 ± 0.7	14.2 ± 0.7	14.4 ± 1.0	14.6 ± 1.3	12.0 ± 1.1
*P*-values	0.11		Two-way interaction 0.20			
Time to reach peak activity (hours)^4^	5.3 ± 0.6	5.5 ± 0.5	4.9 ± 0.6	6.0 ± 0.7	5.8 ± 0.9	4.0 ± 0.7
*P*-values	0.31		Two-way interaction 0.17			
Estrus intensity^5^	95.4 ± 2.1	89.7 ± 2.0	93.2 ± 1.7	96.8 ± 2.0	84.8 ± 3.7	93.0 ± 4.6
*P*-values	0.23		Two-way interaction > 0.20			

^1^Period between GnRH induction of a new follicular wave and estrus onset.

^2^Period between induction of luteolysis (first treatment with CLO) and estrus onset.

^3^Period between estrus onset to re-establishment of normal activity.

^4^Period between estrus onset and the highest/peak value of activity change observed during the estrus event.

^5^Highest/peak value of percentage activity change observed during the estrus event.

### The rate of increase in E_2_ between day 0 and 2 post-induction of luteolysis was predictive of estrus detection

**Figure 5:**
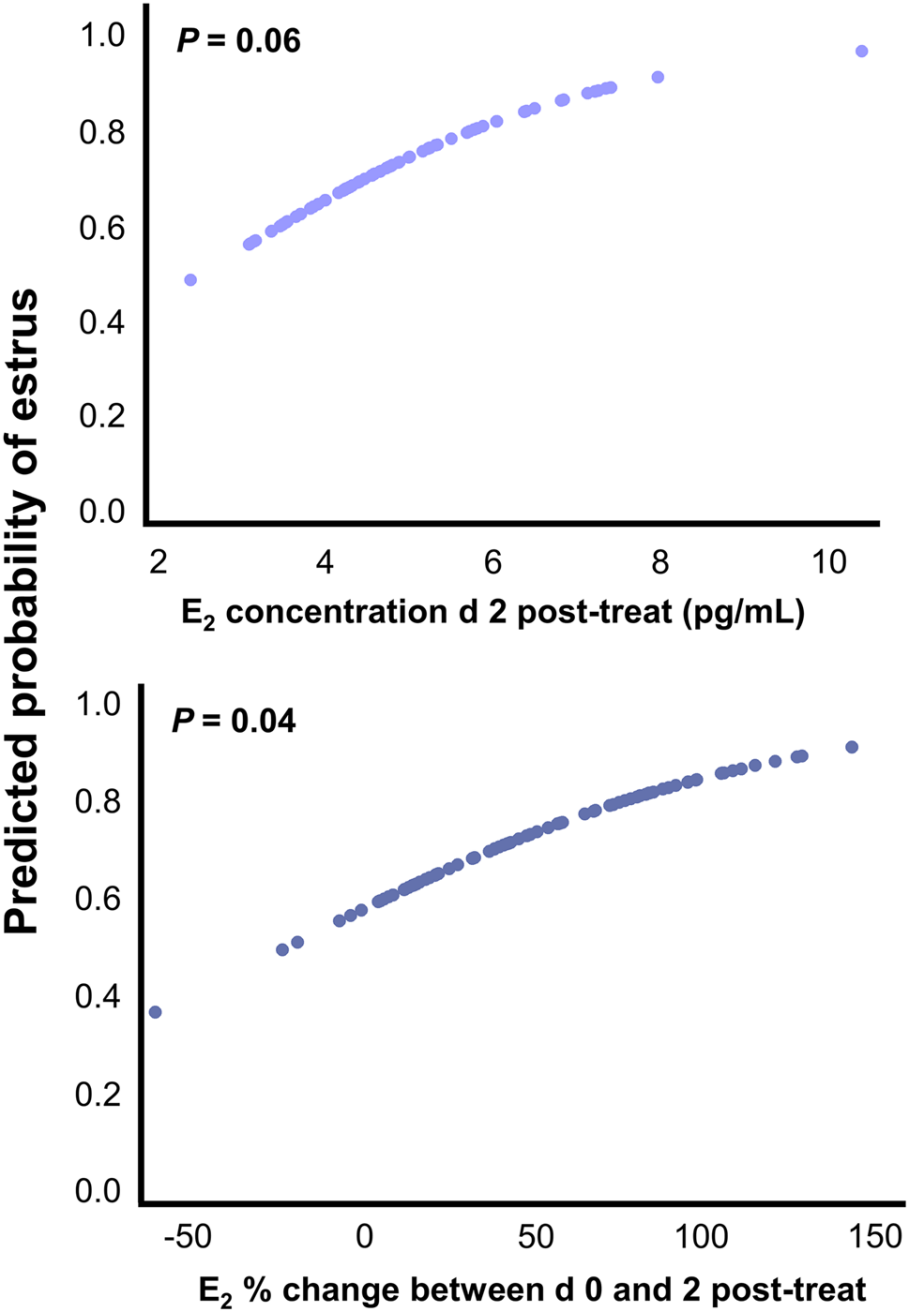
Predicted probability of estrus in relation to 17β-estradiol (E_2_) concentrations (pg/mL) 2 days (d) post-induction of luteolysis (post-treat; **A**) and E_2_ concentrations % change between days 0 and 2 post-induction of luteolysis (**B**). Data shown includes all cows from RAA and Control treatments, regardless of ovulation number (n = 80).

Circulating concentrations of E_2_ 2 days post-induction of luteolysis tended to predict the probability of estrus detection (Fig. 5A). Cows with a greater increase in E_2_ between days 0 and 2 post-induction of luteolysis were more likely to be detected in estrus (Fig. 5B).

## Discussion

Reducing antral age of the pre-ovulatory follicle resulted in smaller, more steroidogenic follicles, as hypothesized. These smaller and younger antral-aged follicles that were 5 days from induction of the follicular wave had a greater increase in E_2_ concentrations between days 0 and 2 post-induction of luteolysis compared with the older follicles that were 7 days from induction of the follicular wave (Fig. 2). Concentrations of E_2_ were not greater 2 days following induced luteolysis in the younger vs older antral aged follicles as our hypothesis anticipated. Earlier induction of final maturation of the pre-ovulatory follicle allowed for a positive correlation between E_2_ and follicle diameter 2 days post-induction of luteolysis compared with delaying luteolysis. LH pulses were not measured in this study, so it remains unclear if differences in E_2_ secretion from these phenotypically different follicles were due to differences in LH pulsatility. We speculate that the differences in increases in E_2_ would most likely be due to antral age-related dysfunction of granulosa cells. Granulosa cell function is imperative for sustained E_2_ secretion during later stages of follicular development^29–31^. Larger follicles have greater numbers of granulosa cells^32^. It would seem plausible that the larger follicle with more granulosa cells would produce more E_2_. Yet, Controls, with larger and more mature follicles, had similar E_2_ concentrations compared with cows with younger antral age 2 days after the start of final maturation. Controls had greater time from wave induction to luteolysis. Follicles from these cows had approximately 2 more days following deviation exposed to P_4_-suppressed LH pulses. The level of P_4_ at the beginning of this period would be produced from at least two corpora lutea (mid- and early-cycle) that were initiated during the implementation of the fertility program Double-Ovsynch. This period of high P_4_ which decreases LH pulsatility has a detrimental effect on granulosa cell function over time^18^. Cows in the RAA group were theoretically only subjected to this high P_4_/low LH pulsatility period for approximately one day if deviation took place on day 4 following wave emergence. Cows in this group had follicles that appeared to be more responsive to LH pulses during the peri/post-luteolytic period and had greater increases in serum E_2_ concentrations compared with Controls.

These outcomes may help to gain a greater understanding of why estrus detection following a complete estrous cycle is a key problem in dairy cattle reproduction today. The antral age of most cows' ovulatory follicles is approximately 11 days, measured from the start of a new mid-cycle wave to ovulation. This is a significant amount of time under the high P_4_/low LH pulse period. Potential granulosa cell dysfunction due to this in addition to exacerbated steroid metabolism due to high milk production^33^ could reduce E_2_ enough to cause true anestrus, and reduce the percentage of cows detected in estrus.

Co-dominance and double ovulation occurred in a similar proportion in RAA and Controls. Lopez et al.^34^ observed a premature rise in the production of E_2_ in cows with co-dominance. The authors suggested that cows with co-dominance had earlier acquisition of cellular characteristics that are concurrent with the dominant follicle phenotype. It appears that in our study Control cows had an earlier increase in E_2_ concentrations due to cows with double ovulations compared with RAA (Fig. 2 and Table 1). These differences could be due to longer periods of post-deviation follicular development in Control cows.

There was no linear relationship between E_2_ concentrations and follicle diameter assessed 2 days post-induction of luteolysis in the Control cows with confirmed single ovulations (Fig. 3). In addition, there was a negative correlation between E_2_ concentrations before induction of luteolysis and dominant follicle diameter 2 days later. This may suggest that some dominant follicles reached maximum E_2_ secretory potential at day 7 of follicular development both functionally (plateau or decrease in E_2_ concentration) and morphologically (decrease in diameter). This again could be associated with progressive loss of function that accompanies follicle aging^18,35^. In the present study, 8/41 (RAA) and 11/39 (Control) cows were not detected in estrus with the automated activity monitoring system. Of these, 5/8 (RAA) and 7/11 (Control) did not ovulate even though all cows had synchronized ovarian function (data not shown). Current timed-AI programs, including Double-Ovsynch, induce ovulation 9.5 days after induction of a new follicular wave with GnRH. Approximately 97% of these follicles will ovulate^13^. This leaves the door open to question the fertility level of some of the oocytes that are “forced-ovulated” with Double-Ovsynch or any other fertility program. Decreased E_2_ secretion due to decreased granulosa cell function could impact oocyte maturation that takes place during proestrus. Estrogen, and mainly estrogen receptor 1 in the granulosa cells, are part of the upstream mechanism that assures oocyte meiotic arrest^36^. Premature meiotic resumption, asynchronous with cytoplastic maturation, as well as early cellular degradation in the follicles would compromise the fertility potential of the oocyte. Reducing antral age resulted in a linear increase of secretory capacity and follicle diameter in cows with single ovulations.

There was no effect of treatment in the steroidogenic potential of the resulting corpora lutea after ovulation, measured as P_4_ concentrations during early diestrus (day 11, 12 and 13 post-estrus). This was unexpected due to differences in diameters of the ovulatory follicles. Data would suggest that smaller ovulatory follicles tend to develop corpora lutea with lower P_4_ at day 18 post-ovulation^37^. Nevertheless, stage of follicle development could be a source of variation when evaluating the impact of ovulatory follicle diameter on subsequent circulating concentrations of P_4_.

It was also hypothesized that cows with the smaller, more steroidogenic, follicles would have greater estrus intensity and longer periods of estrus. Intensity of estrus was greater for cows with younger antral aged follicles at time of induced-luteolysis compared with Controls, as hypothesized (Table 2). But the younger, more steroidogenic, follicles did not translate into cows having longer periods of estrus. Greater estrus intensity around AI, measured as the % change in activity, and the increase in E_2_ in the RAA group could impact oocyte cytoplasm and nuclear maturation^18,38–40^ and result in greater fertility^24^. This could be a key consideration in changing dynamics of follicle development within fertility programs so that a younger and more steroidogenic pre-ovulatory follicle be considered to enhance pregnancy rates/AI.

The effects of double ovulation on estrus characteristics (Table 3) could be due to greater E_2_ concentrations in cases of co-dominance^34^, a hormonal profile reported herein in Control cows (Table 1). Cows with double ovulation had shorter periods to estrus onset, and proestrus length. A greater E_2_ output could have induced an earlier E_2_ peak and onset of sexual receptivity^21^ in these cows. Other key estrus characteristics, such as estrus length and estrus intensity, were not impacted by the occurrence of double ovulations. Based on analyses in the present study, estrus intensity was only weakly correlated with E_2_ concentrations, exclusively in the RAA treatment. Madureira et al.^41^ also reported no correlation between E_2_ concentrations and estrus intensity (r = 0.02) for estrus events occurring after a non-manipulated cycle. However, cows with a high % change in activity (≥ 90%) had greater E_2_ concentrations.

Most fertility programs developed for lactating dairy cows utilize a 7-day period between GnRH and induction of luteolysis with PGF_2α_^8–11^. A 5-day period between induction of follicular wave and induction of luteolysis is also efficient for estrous cycle synchronization with satisfactory pregnancy rates/AI^16,42^. A second dose of cloprostenol sodium can be administered simultaneously, or dinoprost tromethamine can be administered 24 h after the induction of luteolysis, to ensure more cows have regressed corpora lutea^43^. The physiological justification for both strategies would be to induce complete luteolysis in a greater proportion of cows^43^. Incomplete luteolysis prior timed-AI substantially decreases pregnancy likelihood of lactating dairy cows^16,44^. Early developing corpora lutea (i.e., day 5 of development) have refractory characteristics to induced luteolysis^45^, and did not regress following a single injection of PGF_2α_ (dinoprost tromethamine)^46^. Recent evidence also suggested that cows with multiple corpora lutea (regardless of maturity) have decreased luteolytic response and prolonged time to exhibit estrus after administration of a prescribed dose of dinoprost tromethamine^47^. In the present study we utilized the PGF_2α_ analogue pharmaceutical cloprostenol sodium, of longer half-live than dinoprost tromethamine^48^. Two single doses of cloprostenol sodium, given one day apart, effectively resulted in complete luteolysis of refractory 4-day old corpora lutea^49^. An initial double dose, combined with a single dose 24 h later, of cloprostenol sodium resulted in 100% luteolysis rates in this study (Fig. 4). Thus, the treatment regimen utilized for the present study to induce luteolysis was effective on regressing multiple and/or refractory corpora lutea. Santos et al.^16^ reported lower P_4_ concentrations at AI in dairy cows treated with two doses of cloprostenol sodium on days 5 and 6 compared with a single dose of cloprostenol sodium administered on day 7. This evidence could indicate that with appropriate amounts of cloprostenol sodium, a period of 5 days from GnRH to PGF_2α_ could be utilized in fertility programs of lactating dairy cows. Decreasing the period of follicular development by 2 days would result in ovulation of younger follicles, avoiding potential function compromise occurring within a 7-day interval, as described herein. Adjustments in the period from luteolysis to induced ovulation and subsequent AI would likely have to be assessed alongside this strategy.

The average period between induction of luteolysis to estrus expression (proestrus length) was greater for cows in the RAA group compared with Controls (Table 2). The RAA treatment only had an ~ 8-h delay to onset of estrus despite a 2-day difference between wave emergence and induced luteolysis. The difference in proestrus length between treatments was likely associated with increased cumulative E_2_ exposure in Controls, mostly in cows with co-dominance. Sexual receptivity is a process that requires timely exposure to increasing concentrations of E_2_^21,22^. One limitation of the present study was the inability to capture the maximum E_2_ concentrations in relation to estrus onset. A study performed utilizing Holstein–Friesian Irish dairy cows reported that E_2_ concentrations peaked on average 7 h before the increase in activity, or estrus onset^50^. Finally, greater E_2_ concentrations at day 2 post-induction of luteolysis tended to be correlated with greater estrus intensity in RAA, but not Controls. A greater % increase in E_2_ concentrations between day 0 and 2 was associated with greater probability of estrus expression (Fig. 5). Increas

ing estrus expression and intensity during timed-AI programs could be achieved by shortening the period between follicular wave emergence and induced luteolysis.

The proportion of lactating dairy cows detected in estrus tended to be greater in cows with shorter period of follicular development. Contrary to our observation, more dairy cows expressed estrus at timed-AI following a 7-day in comparison to a 5-day Cosynch program^16^. Additionally, beef heifers synchronized with a 7-day CIDR (controlled internal drug releasing P_4_ device) Cosynch also had greater estrus expression than a 5-day CIDR Cosynch^51^. The difference in outcomes compared with the present study could be related to the amount of time cows were allowed to exhibit estrus. In both studies^16,51^, ovulation was induced exogenously about 70 h after luteolysis.

Collectively, these findings would suggest that decreasing the period from follicular emergence to induced luteolysis enhanced the steroidogenic capacity of the pre-ovulatory follicle, despite a smaller diameter. This had a positive impact on estrus characteristics of lactating dairy cows that could be associated with increased fertility.

## Methods

### Experimental units

This project was conducted from July 2021 to September 2021 on a commercial dairy farm, (Nobis Dairy Farm, St. Johns, Michigan, USA). The cows’ owner has provided informed consent to utilize the animals for data collection. Power analyses concluded that n = 34 lactating dairy cows were needed to detect a 2 mm difference in follicular diameter 2 days following induced luteolysis (14 vs 16 mm, SD = 2, α = 0.05, β = 0.2; 5 vs. 7 days of follicular development, respectively). Cows were fed a total mixed ration consisting of corn, wheat, and alfalfa silages, and corn soybean meal-based concentrate formulated to meet nutrient recommendations for high producing lactating dairy cows^52^. The ration was fed once a day in confined free-stall barns with free access to feed and water. The Institutional Animal Care and Use Committee (IACUC) at Michigan State University approved all animal handling and procedures (ID: PROTO202000061). Methods were diligently carried out by authors in accordance with relevant guidelines and regulations. All animal procedures and methods are reported in accordance with ARRIVE guidelines.

### Treatments and model conceptualization

Treatments are described in Fig. 1. Five weekly cohorts totaling n = 91 Holstein cows ranging from 1st to 9th lactation were available and met enrollment criteria. After enrollment n = 8 cows were culled due to farm management reasons. Cows were distributed across parities as follows: 1st (n = 30), 2nd (n = 28), and 3rd+ (n = 25). All cows received Ovsynch beginning 47 to 53 days in milk to pre-synchronize cows to day 0 of the estrous cycle. GnRH was administered to all cows on day 7 of the estrous cycle to initiate a new follicular wave (induced follicular wave emergence). This GnRH induced a new follicular wave 92% of the time when cows were treated with Double-Ovsynch^13^. Cows were then blocked by parity and randomly assigned to one of two treatments. Treated cows were administered 1 mg of cloprostenol sodium (CLO; Boehringer Ingelheim) 5 days following the GnRH-induced new follicular wave to initiate final follicular maturation of a pre-ovulatory follicle with reduced antral age (RAA; n = 41). Control cows received 1 mg CLO 7 days after GnRH to induce final maturation of the pre-ovulatory follicle (Control; n = 42). All cows received an additional 0.5 mg CLO to ensure complete luteolysis 24 h following the initial CLO. Following luteolysis cows were allowed to express natural estrus activity up to 8 days post-induction of luteolysis and received AI from 73 to 83 days in milk (Fig. 1).

All treatments were administered intramuscularly with 1½″ 20 gauge single-use needles in either the semitendinosus or semimembranosus muscles. Cows that did not have functional corpora lutea (P_4_ < 1.0 ng/mL) at time of induction of luteolysis with CLO were removed for analyses. Final analyses included n = 80 cows (RAA, n = 41; Control, n = 39).

### Activity monitoring—estrus characteristics

All cows were equipped with a neck collar fitted with an activity monitoring system (Heatime^®^ Pro+, Allflex Livestock Intelligence). Activity monitoring systems continuously measure acceleration forces and report increases in physical activity to detect behavioral signs of estrus in cattle^23^. Individual cow activity change data were recorded with DataFlow II software (Allflex Livestock Intelligence) in 2-h time increments. Software reports were received via email twice daily. Cows with at least 35% activity increase compared with their past 7-day activity average would be considered in estrus. Onset of estrus was recorded at the time cows went above this threshold. The periods between estrus onset to estrus peak (maximum activity % change for that estrus event), and time to peak, were recorded in hours. Time from estrus onset to normalization of activity (activity below the high activity threshold or estrus length) was also recorded in hours. Maximum percentage increase in activity was considered a measurement of estrus intensity. Cows that were detected in estrus received AI according to am/pm guidelines^53^. Pregnancy outcomes were not considered in the power analyses and were not evaluated in this study.

### Blood samples and hormonal analyses—E_2_ and P_4_ determination

Blood samples were collected to evaluate E_2_ and P_4_ prior to, and 1 and 2 days, following induction of luteolysis, and only P_4_ 11, 12 and 13 days post-estrus. All samples were harvested from the coccygeal vein or artery with serum separation tubes (Venous Blood Collection Tubes: SST, BD Vacutainer). All samples were stored at 4 °C until processing 24 h later. Samples were centrifuged at 2000×*g* for 20 min to allow serum separation. Serum aliquots were frozen at − 18 °C until hormonal analyses. Measurement of E_2_ and P_4_ were performed with radioimmunoassay (RIA; Dr. George Perry, Texas A & M University). Progesterone RIAs were previously described by Engel et. al.^16^. All samples were run in duplicate. Intra- and inter-assay coefficients of variation (CV) were 8.9% and 6.8%, respectively. Assay sensitivity was 0.08 ng/mL. Estradiol RIAs were previously described by Perry and Perry^54^. All samples were run in duplicate and intra- and inter-assay CVs were 1.8% and 4.0%, respectively. Assay sensitivity was 0.5 pg/mL.

### Dominant follicle secretory capacity and complete luteolysis determination

The change over time (rate of increase/decrease) in concentrations of E_2_ and P_4_ were utilized to estimate the dominant follicle secretory capacity, and complete luteolysis, respectively. Concentrations of E_2_ and P_4_ immediately before CLO-induction of luteolysis were considered as baseline concentrations for each cow. A percentage change (*% change* = (*observed* − *baseline/baseline*) × *100*) was calculated between day 0 and 1, day 0 and 2, and day 1 and 2, for both E_2_ and P_4_. Additionally, complete luteolysis was defined as either P_4_ < 1 ng/mL 2 days after induction of luteolysis, or a reduction of > 500% between induction of luteolysis and 2 days later.

### Ultrasonography—follicle diameter and confirmation of ovulation

Linear array ultrasonography was used to map ovaries and describe ovarian structures (MyLab Gamma, Esaote). Luteal and follicular structures > 8 mm in diameter were measured and mapped 2 days following induction of luteolysis. All measurements were performed using built-in calipers. The first diameter measurement was measured horizontally at the greatest width and the second diameter was measured perpendicular to the first diameter, at the greatest height. The final follicular diameter was reported in mm as the average of both measurements. Cows were reassessed with ultrasound at either 8 (RAA) or 6 (Control) days after induction of luteolysis to determine the appearance of newly formed corpora lutea and ovulation (Fig. 1). Two cows in RAA failed to ovulate following estrus detection. Cows with double ovulation were removed from all analyses that included follicle diameter measurements (RAA, n = 11; Control, n = 7).

### Statistical analyses

All statistical analyses were performed utilizing SAS 9.4. Binary variables were analyzed with a generalized linear mixed model fitted with PROC GLIMMIX. Differences in proportions were tested using the chi-square test of independence. Treatment and parity were included in the model as fixed effects.

Continuous variables were analyzed utilizing PROC MIXED. Concentrations of E_2_ and P_4_ at days 0, 1 and 2 were estimated with PROC MIXED and with the REPEATED statement to account for measurements performed over time. These analyses included two-way and three-way interactions between time, treatment, and double ovulation. An auto-regressive model was also utilized (AR1). All models included treatment and parity as fixed effects. Double ovulation was specified as a fixed effect in analyses that included all cows. Interaction terms of treatment and double ovulation were maintained in the models when *P* ≤ 0.20. Degrees of freedom were estimated utilizing Kenward-Roger approximation. Differences within fixed effects were sliced using the LSMEANS statement and adjusted with Tukey–Kramer in the event of multiple comparisons. Linear regression between follicle diameter and E_2_ concentrations at 2 days after induction of luteolysis was estimated with PROC REG. Pearson correlation coefficients between two continuous variables were estimated with PROC CORR. Predicted probability of estrus as a function of E_2_ concentrations and E_2_ concentrations % change was estimated with PROC LOGISTIC. The significance level was set at α ≤ 0.05 for all analyses. *P*-values were rounded to nearest hundredth.

## Data Availability

Data is available upon email request to corresponding author (pursleyr@msu.edu).
